# Targeted complement inhibition using bispecific antibodies that bind local antigens and endogenous complement regulators

**DOI:** 10.3389/fimmu.2024.1288597

**Published:** 2024-05-16

**Authors:** Haiyu Wang, Fleur S. van de Bovenkamp, Douwe J. Dijkstra, Leoni Abendstein, Nicole V. Borggreven, Jos Pool, Rob Zuijderduijn, Christoph Gstöttner, Kyra A. Gelderman, Timon Damelang, Gestur Vidarsson, Anna M. Blom, Elena Domínguez-Vega, Paul W. H. I. Parren, Thomas H. Sharp, Leendert A. Trouw

**Affiliations:** ^1^Department of Immunology, Leiden University Medical Center, Leiden, Netherlands; ^2^Department of Cell and Chemical Biology, Leiden University Medical Center, Leiden, Netherlands; ^3^Center for Proteomics and Metabolomics, Leiden University Medical Center, Leiden, Netherlands; ^4^Sanquin Diagnostic Services, Amsterdam, Netherlands; ^5^Sanquin Research, Department of Experimental Immunohematology, Amsterdam, Netherlands; ^6^Department of Biomolecular Mass Spectrometry and Proteomics, Utrecht Institute for Pharmaceutical Sciences and Bijvoet Center for Biomolecular Research, Utrecht University, Utrecht, Netherlands; ^7^Department of Translational Medicine, Section of Medical Protein Chemistry, Lund University, Malmö, Sweden; ^8^Gyes BV, Utrecht, Netherlands

**Keywords:** complement, antibobies, inhibition, targeted, autoimmunity

## Abstract

Complement activation protects against infection but also contributes to pathological mechanisms in a range of clinical conditions such as autoimmune diseases and transplant rejection. Complement-inhibitory drugs, either approved or in development, usually act systemically, thereby increasing the risk for infections. We therefore envisioned a novel class of bispecific antibodies (bsAbs) which are capable of site-directed complement inhibition by bringing endogenous complement regulators in the vicinity of defined cell surface antigens. Here, we analyzed a comprehensive set of obligate bsAbs designed to crosslink a specific target with either complement regulator factor H (FH) or C4b-binding protein (C4BP). The bsAbs were assessed for their capacity to inhibit complement activation and cell lysis in an antigen-targeted manner. We observed that the bsAbs inhibited classical, lectin, and alternative pathway complement activation in which sufficient endogenous serum FH and C4BP could be recruited to achieve local inhibition. Importantly, the bsAbs effectively protected antigen-positive liposomes, erythrocytes, and human leukocytes from complement-mediated lysis. In conclusion, localized complement inhibition by bsAbs capable of recruiting endogenous human complement regulators (such as FH or C4BP) to cell surfaces potentially provides a novel therapeutic approach for the targeted treatment of complement-mediated diseases.

## Introduction

The complement system is important for the clearance of pathogens, thereby providing protection against infection. It consists of three pathways that are initiated by e.g. antibodies (Abs) or sugar structures on pathogens, but that all result in the formation of C3 and C5 convertases. The action of these convertases produces the anaphylatoxins C3a and C5a that mediate inflammation and the formation of membrane attack complexes (C5b-9), which mediate cell lysis. Undesired, misdirected or excessive complement activation contributes to cell and tissue damage. This is the case in several (auto)antibody-driven diseases that predominantly activate the classical pathway (1), for example in autoimmune diseases (anti-collagen Abs in arthritis (2), anti-glomerular basement membrane Abs in nephritis (3), anti-acetylcholine receptor Abs in myasthenia gravis (4)) and transplant rejection (anti-HLA Abs) (5). Additionally, lectin pathway and alternative pathway-driven complement activation can result in severe tissue damage, for example in atypical hemolytic uremic syndrome or age-related macular degeneration (6).

To reduce complement-mediated damage in these diseases, a number of complement-inhibitory drugs have been developed, of which a few have already made their way into the clinic and were approved for the treatment of several complement-mediated diseases (7–11). Importantly, these therapeutics inhibit the complement system systemically. Since this is associated with increased risk for infections and need for high dosing, we instead aimed for locally targeted complement inhibition by developing bispecific antibodies (bsAbs), where one Fab arm binds to a tissue-specific target and the other Fab arm binds to a human endogenous complement inhibitor.

Next to a set of membrane-bound complement inhibitors, such as complement receptor 1 (CR1, CD35), membrane cofactor protein (MCP, CD46), decay-accelerating factor (DAF, CD55), and membrane attach complex-inhibitory protein (MAC-IP, CD59), several fluid-phase complement inhibitors circulate in the blood to prevent unwanted complement activation (12). The most well-known fluid-phase inhibitors, factor H (FH), factor I (FI), and C4b-binding protein (C4BP), collectively limit autoactivation of complement in the circulation (13). C4BP is the main fluid-phase regulator of the classical and lectin pathways. It binds to C4b, displacing C2a, thereby enhances decay of C3 convertase, and in addition acts as a cofactor for C4b cleavage by FI (14). FH is the main fluid-phase regulator of the alternative pathway, which binds to C3b, displacing Bb, and also acts as a cofactor for C3b cleavage by FI (14).

In this study we developed a comprehensive set of bsAbs binding FH or C4BP as well as specific (auto)antigens. BsAbs were generated by controlled Fab-arm exchange (15), and to prevent complement activation and immune activation by the bsAbs themselves, we introduced LALAPG mutations in the Fc region that have been shown to block C1q and FcγR binding (16). Our results indicate that locally targeted complement inhibition is feasible using the bsAb format that enforces colocalization of human endogenous complement inhibitors and specific (auto)antigens, potentially representing a promising novel therapeutic approach for the treatment of complement-mediated diseases.

## Materials and methods

### Antibody sequences

The variable heavy (VH) and variable light (VL) sequences of anti-FH (OX-24) and anti-C4BP (3B9D6) were obtained by sequencing hybridomas (see below). Anti-biotin (17), anti-CD20 (rituximab, DrugBank Accession Number DB00073), anti-CD52 [alemtuzumab (18)], anti-2,4-dinitrophenol (DNP) (19), anti-HIV gp120 [b12 (20)], and anti-HLA I [W6-32 (21)] VH and VL sequences were extracted from literature or an online database (DrugBank).

### 3B9D6 and OX-24 hybridomas

Hybridomas producing Abs directed against human FH were acquired from the European Collection of Authenticated Cell Cultures (ECACC, OX-24). Hybridomas producing Abs directed against human C4BP were acquired from Podiceps BV, Utrecht, the Netherlands (3B9D6). Hybridoma cells were cultured in Roswell Park Memorial Institute (RPMI) 1640 medium (Gibco) supplemented with penicillin, streptomycin, 10% fetal calf serum (FCS), 2 mM L-glutamine (all Gibco), and 50 µM β-mercaptoethanol (Sigma-Aldrich) at 37°C in 5% CO_2_.

### Sequencing

RNA was isolated from hybridoma cells using TRIzol reagent (Thermo Fisher Scientific), chloroform, isopropanol, and ethanol (all Merck) according to the instructions provided by the manufacturer. Next, SMART cDNA synthesis was performed using PrimeScript Reverse Transcriptase (Takara), and VH and VL gene products were amplified by 5’-RACE polymerase chain reaction (PCR). The VH and VL PCR products were treated with Taq polymerase (Promega) for 10 minutes at 37°C and afterwards purified from excised bands using the Zymoclean gel DNA recovery kit (Zymo Research). Subsequently, the VH and VL products were cloned into pGEM-T Easy vectors (Promega). The vectors were used for transformation of JM109 competent cells (Promega) by heat shock. The transformed cells were cultured on LB agar plates supplemented with 100 µg/mL ampicillin (Sigma-Aldrich) and after overnight incubation at 37°C, multiple single colonies were picked and grown overnight in LB medium containing ampicillin. From the cultures, plasmids were isolated using the QIAprep spin miniprep kit (Qiagen). The plasmids were sequenced by the Leiden Genome Technology Center (LGTC) to obtain the VH and VL sequences.

### DNA constructs

DNA constructs consisting of the HindIII restriction site, a Kozak sequence (GCCGCCACC), a signal peptide (MEFGLSWVFLVALLRGVQC), the VH/VL sequence, and the ApaI restriction site (in case of VH) or the BsiWI restriction site (in case of VL) were designed. DNA constructs were ordered as a DNA fragment or in a DNA vector (GeneArt – Thermo Fisher Scientific). DNA constructs were digested with HindIII and ApaI or BsiWI and ligated into a pcDNA3.3 expression vector containing the constant domain of human IgG1 (IGHG1*03) or κ (IGKC). Depending on the specificity, different mutations were introduced. Anti-CD20 and anti-CD52 were kept wild-type (IgG1κ). Anti-FH and anti-C4BP were designed with the F405L mutation, whereas anti-DNP, anti-biotin, and anti-HLA I were designed with the K409R mutation (mutations necessary for Fab-arm exchange, see below). Anti-HIV was designed with either the F405L mutation or the K409R mutation. All IgG Abs except anti-CD20 and anti-CD52 contained LALAPG mutations to make their Fc functionally inactive (16). Anti-Vel IgM was previously cloned in pcDNA3.1 (22).

### Transfection

For antibody production, heavy and light chain containing vectors were used for transient co-transfection of Expi293F cells with ExpiFectamine, Opti-MEM, and Expi293 expression medium (Thermo Fisher Scientific) according to the instructions provided by the manufacturer. After 5 days of culture, supernatants containing the Abs were harvested and filtered.

### Antibody purification (protein A)

For antibody purification, filtered supernatants were loaded onto a column packed with protein A resin (GenScript). After washing the column with PBS, Abs were eluted with 20 mM glycine pH 2.8 and neutralized with 1 M Tris HCl pH 9.5. Abs were concentrated and buffer exchanged to PBS using Ultra Centrifugal filter units (Merck). Concentrations of purified Abs were determined by NanoDrop.

### Fab-arm exchange

The generation of bsAbs was done by Fab-arm exchange as previously described (15). The complement regulator (FH/C4BP)-binding parental Abs had the F405L-LALAPG mutations and the target (DNP/biotin/HLA I)-binding parental Abs had the K409R-LALAPG mutations. The anti-HIV arm served as a non-binding arm (isotype control) to preserve the bsAb architecture. Throughout this article we refer to a bsAb using AAA x BBB, with AAA being one of the Abs bearing the F405L-LALAPG mutations and BBB being one of the Abs bearing the K409R-LALAPG mutations. The quality of the bsAbs was confirmed via bispecificity ELISA and mass spectrometry (see below).

### Gel electrophoresis

Parental and bsAbs were analyzed by SDS-PAGE by loading 5 µg IgG on precast 4-15% Tris-Glycine gels (BIO-RAD), visualized with Coomassie Blue (InstantBlue).

### Bispecificity ELISA

BsAbs were tested for simultaneous binding to both antigens in a bispecificity ELISA. MaxiSorp plates (Thermo Scientific, Nunc, 430341) were coated with 50 µL/well BSA-DNP (10 µg/mL, Biosearch Technologies, D-5050-10, conjugation ratio 13) in coating buffer (0.1 M Na_2_CO_3_/NaHCO_3_ pH 9.6) for 1 hour at 37°C. After every incubation, plates were washed three times with PBS/0.05% Tween 20. Plates were blocked with 100 µL/well PBS/1% bovine serum albumin (BSA) for 1 hour at 37°C. After washing, plates were incubated with 50 µL/well (bs)Abs (concentrations are indicated in figures) diluted in PBS/0.05% Tween 20/1% BSA (PTB buffer) for 1 hour at 37°C. Next, 1 µg/mL FH (CompTech, A137) or C4BP (CompTech, A109) diluted in PTB buffer was added. FH was detected using goat anti-human FH (Quidel, A312) and HRP-labeled rabbit anti-goat Ig (Dako, P0449), and C4BP was detected using rabbit anti-human C4BP (generated in-house by group of Anna M. Blom, Malmö, Sweden) and HRP-labeled goat anti-rabbit Ig (Dako, P0448), all diluted in PTB buffer. Plates were developed by incubating with 50 µL/well ABTS (Merck, A1888-5G) containing 1:2000 diluted H_2_O_2_ (Merck, 1072090250), and read at 415 nm using a microplate reader (BIO-RAD iMark).

### Mass spectrometry

Intact mass analysis of parental and bsAbs was performed using sheathless capillary electrophoresis (CESI 8000 instrument, Sciex) hyphenated to an Impact QTOF mass spectrometer (Bruker Daltonics). A bare-fused silica capillary containing a porous tip (91 cm x 30 µm ID, Sciex) was used for the analyses. Prior to the experiments, the capillary was coated with polyethylenimine (Gelest) following the protocol described by Sciex (23). As background electrolyte (BGE) 10% acetic acid was employed. Prior to each run, the capillary was flushed for 3 minutes at 100 psi (forward and reverse pressure) with the BGE. Before analysis, samples were buffer exchanged to 10 mM acetate adjusted with ammonium acetate to pH 3.1, using 30 kDa MWCO filters (Vivaspin, 3 cycles of 10000xg at 4°C). Samples were injected hydrodynamically by applying 2.5 psi for 15 seconds. Separation was carried out applying a voltage of -20 kV at 20°C. The mass spectrometer was operated in positive ionization mode using a capillary voltage of 1100 V, a drying gas temperature of 120°C, and a flow of 1.2 L/minute. An in-source CID energy of 100 eV was used for declustering. To further enhance declustering, the quadrupole and collision cell energy were set at 5.0 and 20.0 eV, respectively. For data analysis, the Data Analysis software (Bruker) was used. To determine the intact mass of the (bs)Abs, the mass spectra were deconvoluted using the Maximum Entropy deconvolution algorithm. A baseline subtraction of 0.8 points was applied on the obtained zero deconvoluted spectra.

### Complement activation assay

BsAbs were functionally tested in plate-bound complement activation assays. To focus on the classical pathway, MaxiSorp plates were coated with 50 µL/well of a mixture of IgG (10 µg/mL, IVIg, Sanquin) or IgM (1 µg/mL, Merck, 401799) and BSA-DNP (10 µg/mL) or biotinylated BSA-DNP (10 µg/mL, biotinylated in-house) in coating buffer for 1 hour at 37°C. After every incubation, plates were washed three times with PBS/0.05% Tween 20. Plates were blocked with 100 µL/well PBS/1% BSA for 1 hour at 37°C. Samples containing 5 µg/mL (or a titration from 0.1 to 10 µg/mL) bsAbs, 10 µg/mL FH or C4BP, and 1% normal human serum (NHS) in RPMI were preincubated for 30 minutes at 4°C. After washing, plates were incubated with 50 µL/well samples for 1 hour at 37°C. Next, FH and C4BP were detected as described above for the bispecificity ELISA (using either goat anti-human FH and HRP-labeled rabbit anti-goat Ig, or using rabbit anti-human C4BP and HRP-labeled goat anti-rabbit Ig), and C5b-9 was detected using mouse anti-human C5b-9 (Dako, M0777) and HRP-labeled goat anti-mouse Ig (Dako, P0447), all diluted in PTB buffer. Plates were developed by incubating with 50 µL/well ABTS containing 1:2000 diluted H_2_O_2_, and read at 415 nm using a microplate reader.

To focus on the lectin pathway, a similar assay was performed, but plates were coated with a mixture of acetylated human serum albumin (HSA,10 µg/mL, acetylated in-house) and BSA-DNP. In addition to NHS, C1q- and factor B-depleted serum were used, all at 3%. To focus on the alternative pathway, plates were coated with a mixture of lipopolysaccharide (LPS,10 µg/mL, Sigma-Aldrich, L2012-10MG) and BSA-DNP. Besides, a higher percentage of NHS (10%) was used and samples were prepared in RPMI-MgEGTA (5 mM MgCl_2_, 10 mM EGTA).

### Liposome preparation

Liposomes decorated with two antigens, mCD52 (peptide with the amino acid sequence TSSPSAD, which is a CD52 mimotope; synthesized by Aimee Boyle, Leiden Institute of Chemistry, Leiden University, Leiden, the Netherlands) and DNP, encapsulating sulforhodamine B (20 mM; Sigma-Aldrich, S1402) in PBS were produced. Lipid films were composed of dimyristoylphosphatidylcholine (DMPC), dimyristoylphosphatidylglycerol (DMPG), cholesterol, DNP-cap-PE and mCD52-cholesterol (44:5:49:1:1 mol%). Liposomes were prepared as described previously (24–26). All lipids, except mCD52-cholestrol, were purchased from Avanti Polar Lipids.

### Liposome assay

The effect of the bsAbs (anti-FH/C4BP x anti-DNP and controls) on the classical pathway was analyzed in a fluorescence-based complement activation assay using liposomes. Therefore, purified liposomes displaying CD52 and DNP antigens, and encapsulating sulforhodamine B were incubated with bsAbs (50 µg/mL; 333.3 nM final concentration), NHS (1% v/v final concentration), and with or without either FH (100 µg/mL; 645.2 nM final concentration) or C4BP (100 µg/mL; 200 nM final concentration) for 1 hour at 4°C, before transferring them to a microplate (Merck, CLS4514). Sulforhodamine B fluorescence was measured using a CLARIOstar microplate reader (BMG LABTECH) with an excitation wavelength of 565 nm and emission wavelength of 585 nm at 21°C. After measuring the fluorescence intensity for 15 minutes, anti-CD52 wild-type (50 µg/mL; 333.3 nM final concentration) was added and fluorescence intensity was measured for another 30 minutes. For the positive control, liposomes were incubated with NHS (without bsAbs) before anti-CD52 wild-type was added. For the negative control, liposomes were incubated with NHS (without bsAbs) and no anti-CD52 wild-type was added.

### Hemolytic assay

To study the effect of the targeted bsAbs on activity of the classical pathway, (rabbit anti-sheep) antibody-sensitized sheep red blood cells (Sanquin) were washed three times with PBS and biotinylated (Thermo Scientific, 21331) for 30 minutes at room temperature. After washing two times with PBS, cells (10 million/well) were incubated with 10 µg/mL (or a titration from 0.1 to 10 µg/mL) bsAbs (anti-FH/C4BP x anti-biotin and controls) with or without 10 µg/mL FH or C4BP diluted in PBS for 30 minutes at room temperature. After washing, cells were incubated with 0.5% NHS in RPMI for 1 hour at 37°C. For the positive control, cells were incubated with water to get 100% lysis. Cells were centrifuged and supernatants were transferred to MaxiSorp plates, which were read at 405 nm using a microplate reader.

To study the effect of the targeted bsAbs on activity of the alternative pathway, a similar assay was performed, but rabbit red blood cells (which lack endogenous FH binding, Sanquin) were used and incubated with 5% NHS in RPMI-MgEGTA.

The effect of the targeted bsAbs on activity of the classical pathway was also studied in a completely human system. A similar assay was performed, but human red blood cells (from a blood group A positive donor, Sanquin) treated with bromelin (Sanquin) were biotinylated and incubated with 5% serum (from an AB donor, Sanquin) and 0.2 µg/mL anti-Vel IgM (Sanquin) in RPMI.

### CDC assay

Ramos cells (originally from ATCC) were plated at 100,000 cells/well (V-bottom microplate, Greiner Bio-One, 651101). Cells were incubated with 100 µL/well samples containing 0.3 µg/mL anti-CD20 wild-type with or without 20 µg/mL bsAbs (anti-FH x anti-HLA I and controls) with or without 10 µg/mL FH for 30 minutes. After every incubation, cells were washed with 150 µL/well PBS/1% FCS. Cells were incubated with 100 µL/well 10% NHS in RPMI (for FH detection in the presence of 50 µg/mL eculizumab) for 45 minutes at 37°C. Cell viability was measured by adding 100 µL/well 7-AAD (BD Pharmingen, 559925) 1:200 diluted in PBS/1% FCS. FH was detected using mouse anti-human FH (1:100, OX-23, generated in-house) and goat anti-mouse IgG AF488 (1:50, Life Technologies, A11001). Cells were measured using a FACSCanto flow cytometer (BD Biosciences).

A similar assay was performed with thawed peripheral blood mononuclear cells (PBMCs, isolated from healthy donors using Ficoll) instead of cultured Ramos cells. For PBMCs, 1 µg/mL anti-CD52 wild-type instead of anti-CD20 wild-type was used to kill cells, and a higher concentration of bsAbs (30 µg/mL) was used focusing on FH as the inhibitor, as B cells are known to have some interaction with C4BP.

In a similar setting using PBMCs we verified the capacity of our bi-specifics to capture FH or C4BP on one cell type (B-cells) and not on another (T cells). For that purpose PBMCs were incubated with bsAbs comprising one arm rituximab and the other arm either anti-FH, anti-C4BP or anti-HIV with all the other relevant controls. Next NHS was added and accumulation of FH or C4BP was verified staining for FH or for C4BP together with staining for CD19 as a marker for B cells and CD3 as a marker for T cells. Further details are shown in **Supplementary Figure 8**.

## Results

### Generation of bispecific antibodies

**Figure 1:**
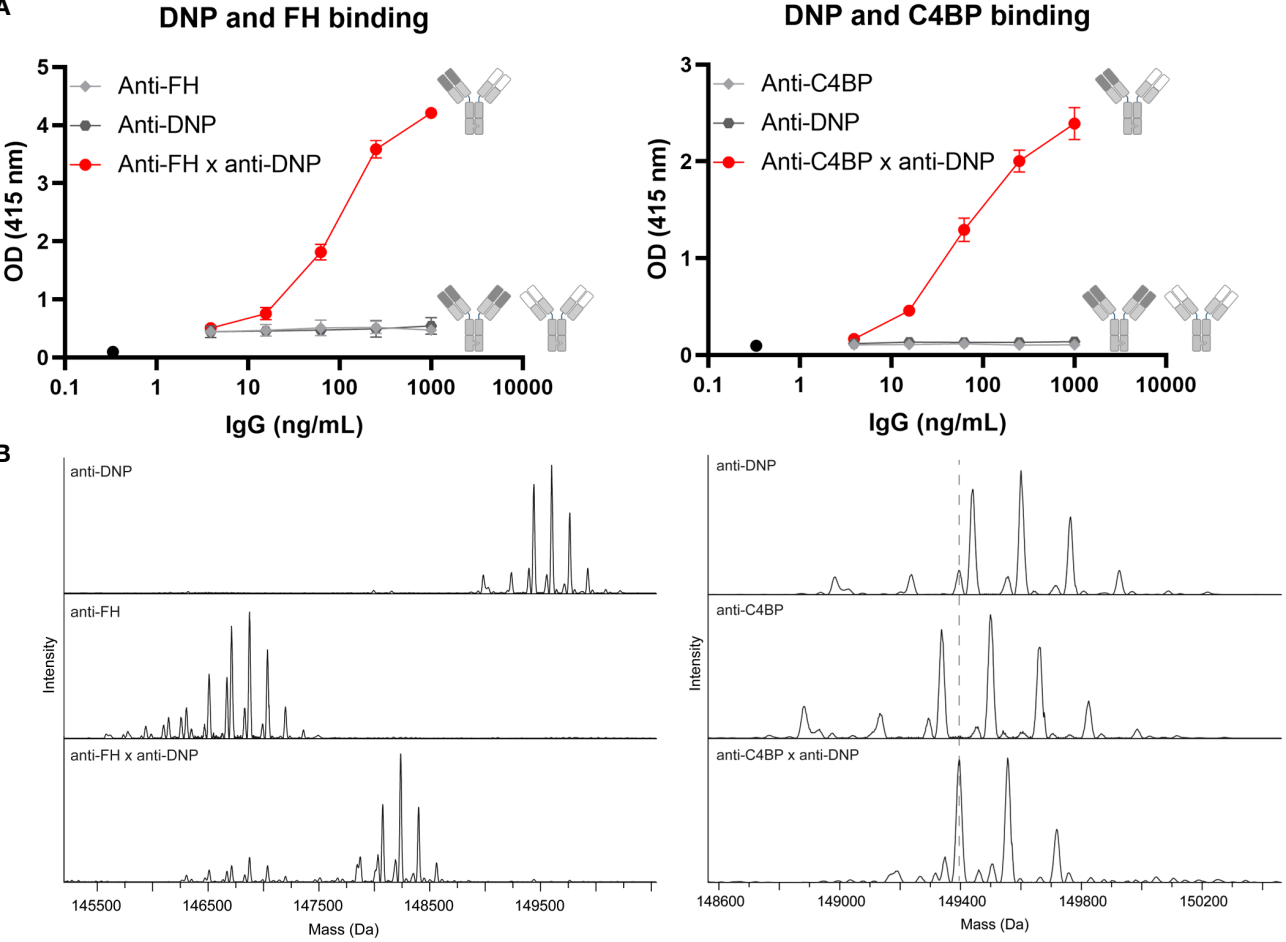
Successful generation of bispecific antibodies. **(A)** BsAbs were tested for simultaneous binding to both antigens in a bispecificity ELISA. Plates were coated with BSA-DNP and incubated without Abs (black circle) or with anti-FH (left, light gray diamonds), anti-C4BP (right, light gray diamonds), anti-DNP (dark gray hexagons) parental Abs, or bsAbs (red circles). FH or C4BP was added and these inhibitors were detected. Data of one representative experiment, run in triplicates, out of at least three experiments are presented. **(B)** Intact masses of parental and bsAbs were determined by mass spectrometry. All schematics have been generated using Biorender.

We generated a comprehensive set of bsAbs to target and accumulate complement inhibitors FH or C4BP to specific locations. Monospecific Abs were generated bearing LALAPG mutations in the Fc region to block C1q and FcγR binding (16), making them unable to elicit Fc-mediated effector functions. In addition, the Fc region contained additional complementary mutations that allow the generation of bsAbs using the procedure of controlled Fab-arm exchange (15). We generated pure fractions of bsAbs (**Supplementary Figure 1**) that simultaneously bind to a site-specific target (e.g., DNP as a model antigen) as well as to the endogenous complement regulators FH or C4BP, whereas the parental monospecific Abs do not bind to these targets (**Figure 1A**). Using mass spectrometry, we confirmed efficient Fab-arm exchange with the masses of the bsAbs showing an average between the masses of the parental monospecific Abs (**Figure 1B**). Although for some of the parental monospecific Abs the range of masses overlapped (e.g., ranging from 149,338 to 149,985 Da for anti-C4BP and from 149,439 to 150,086 Da for anti-DNP), we were able to show the presence of the bsAb based on the average masses (**Figure 1B**, right). Minimal amounts of parental monospecific Abs were observed in the bsAb preparations, indicating that we successfully generated highly pure fractions of bsAbs. For the subsequent functional analyses, all bsAbs were compared with control bsAbs in which either the target antigen-binding arm or the complement regulator-binding arm was replaced by an antibody arm binding HIV gp120 (b12) as an irrelevant antigen.

### Targeted bispecific antibodies that bind endogenous complement inhibitors decrease complement activation

**Figure 2:**
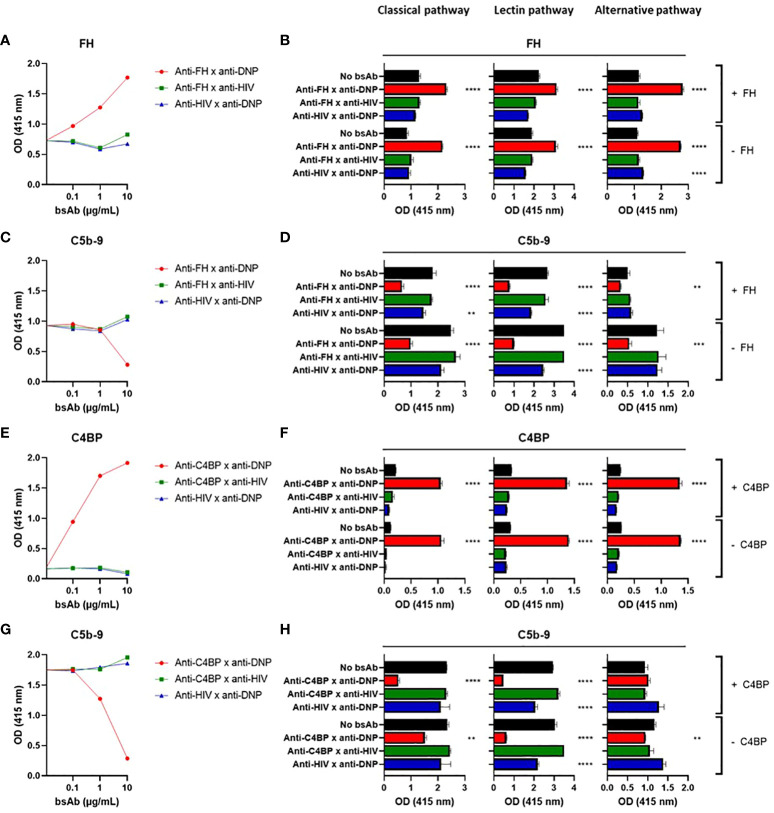
Targeted bispecific antibodies that bind endogenous complement inhibitors decrease complement activation in situ. BsAbs were functionally tested in plate-bound complement activation assays. Plates coated with BSA-DNP and IgG (classical pathway), acetylated HSA (lectin pathway), or LPS (alternative pathway) were incubated with a titration of anti-FH x anti-DNP **(A, C)**, 5 µg/mL anti-FH x anti-DNP **(B, D)**, a titration of anti-C4BP x anti-DNP **(E, G)**, or 5 µg/mL anti-C4BP x anti-DNP **(F, H)** or control bsAbs, with NHS with (+) or without (-) exogenous FH **(A–D)** or C4BP **(E–H)**. FH **(A, B)**, C4BP **(E, F)**, and C5b-9 **(C, D, G, H)** were detected. In **(A, C, E, G)** IgG-coated plates were used. Bars indicate means and error bars indicate standard deviations of one representative experiment out of at least three experiments. One-way ANOVA compared to no bsAb, **P < 0.01; ***P < 0.001; ****P < 0.0001.

Next, we tested the functional effects of our bsAbs in plate-bound complement activation assays. First we verified that we can activate each of the three individual complement pathways in plate bound assays (**Supplementary Figures 2A, B**). A specific target (DNP) was coated to allow binding of the bsAbs, together with IgG to activate the classical pathway, acetylated HSA to activate the lectin pathway, or LPS to activate the alternative pathway of the complement system. Upon incubation with NHS, an increase in bound FH was detected for the bsAb binding both FH and DNP, whereas for the control bsAbs (binding either FH or DNP) FH binding did not increase (**Figures 2A, B**). This increase in FH binding was associated with a decrease in C5b-9 (**Figures 2C, D**), showing that the bsAb binding both FH and DNP indeed inhibits complement activation. Increasing the FH concentration by adding exogenous FH to NHS (which at 1% contains ~3-5 µg/mL FH) was not necessary and did not improve the complement-inhibiting effect, indicating that FH endogenously present in human serum is sufficient to mediate the desired effects (**Figure 2D**).

For the bsAb binding both C4BP and DNP, an increase in bound C4BP was detected upon incubation with NHS, whereas for the control bsAbs (binding either C4BP or DNP) C4BP binding did not increase (**Figures 2E, F**). This increase in C4BP binding was associated with a decrease in C5b-9 (**Figures 2G, H**), showing that similarly to the FH results above, the bsAb binding both C4BP and DNP also inhibits complement activation. Increasing the C4BP concentration by adding exogenous C4BP to NHS (which at 1% contains ~2 µg/mL C4BP) was not necessary but, in contrast to FH, did improve inhibition of the classical but not of the alternative or lectin pathways (**Figure 2H**). The complement inhibition mechanism of action is shown in **Supplementary Figure 7A**.

For the classical pathway, we repeated our experiments using another more potent initiating molecule, namely IgM. We obtained very similar results, confirming that the bsAbs binding both FH and DNP or C4BP and DNP (**Supplementary Figures 3A-D**) capture FH or C4BP, respectively, thereby increasing their local concentration, and decrease C5b-9 formation and thus inhibit complement activation. For the lectin pathway, we repeated our experiments using C1q- or factor B-depleted serum and observed clear inhibition (**Supplementary Figure 4**), indicating that the observed inhibition is not (only) classical or alternative pathway dependent but driven by lectin pathway activity. When exchanging the DNP antigen with biotin and using other bsAbs that bind to both FH/C4BP and biotin, the results were similar (**Supplementary Figures 5A-C**), indicating that the complement-inhibiting effect observed for the set of bsAbs is a general phenomenon and is not dependent on the specific target.

In summary, we observed that in plate-bound assays the bsAbs inhibited complement activation initiated by the classical, lectin, and alternative pathway. The concentrations of endogenous FH and C4BP in serum were sufficient to mediate local inhibition.

### Targeted bispecific antibodies decrease lysis of liposomes

**Figure 3:**
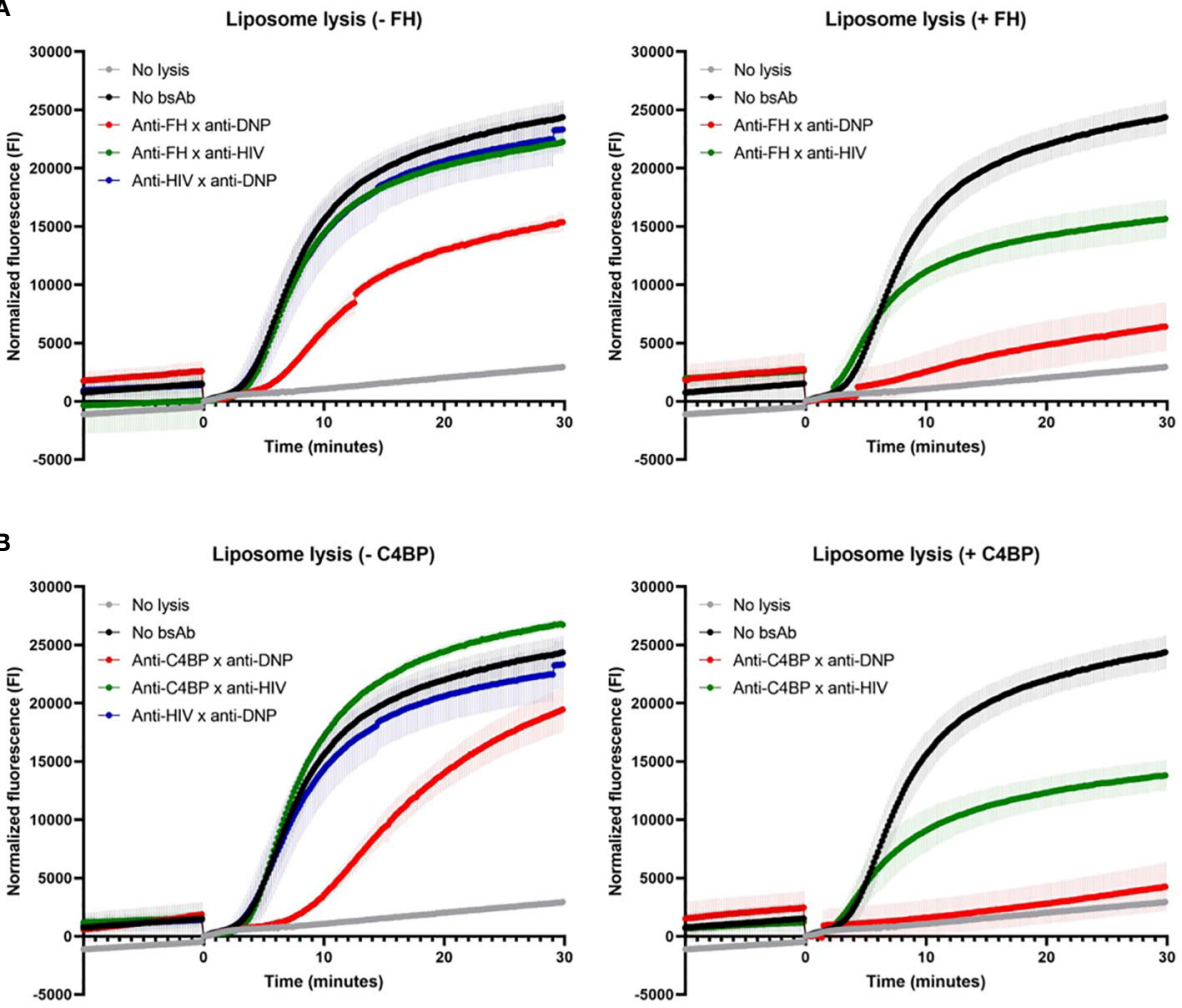
Targeted bispecific antibodies decrease lysis of liposomes. The effect of the bsAbs on the classical pathway was analyzed in a fluorescence-based complement activation assay using liposomes. Liposomes containing DNP and CD52, filled with sulforhodamine B, were incubated with anti-FH x anti-DNP **(A)** or anti-C4BP x anti-DNP **(B)** or control bsAbs, with (+, right) or without (-, left) exogenous FH/C4BP and NHS. After 1 hour, sulforhodamine B fluorescence was measured. After 10 minutes (0 minutes in graphs), anti-CD52 wild-type was added and fluorescence was measured for another 30 minutes (indicated by the arrow). Data of one representative experiment out of at least three experiments are presented.

We next tested whether the bsAbs were also able to protect liposomes, acting as fluid-phase cell mimetics, from complement-mediated lysis. As shown in the cartoon (**Supplementary Figure 7B**). liposomes displaying CD52 and DNP antigens, and encapsulating sulforhodamine B, were incubated with the bsAbs binding both FH/C4BP and DNP, and NHS with or without exogenous FH or C4BP. Upon adding anti-CD52 Abs to activate the classical pathway of the complement system, lysis of liposomes was clearly decreased by the bsAb binding both FH and DNP and not by the control bsAbs (binding either FH or DNP) (**Figure 3A**, left). Adding exogenous FH to NHS already decreased liposome lysis in the presence of the control bsAb binding only FH, but importantly a near complete block in liposome lysis was obtained in the presence of the bsAb binding both FH and DNP (**Figure 3A**, right). This shows that while fluid-phase FH can decrease liposome lysis, our bsAb is much more effective in protecting liposomes from complement-mediated lysis by targeting more efficiently FH to the liposomes, thereby providing important proof of concept. Very similar results were obtained with the bsAb binding both C4BP and DNP, which with adding exogenous C4BP to NHS almost completely prevented liposome lysis (**Figure 3B**). Overall, the data indicate that the bsAbs protect liposomes, acting as fluid-phase cell mimetics, from complement-mediated lysis.

### Targeted bispecific antibodies protect erythrocytes from complement-mediated lysis

To test the bsAbs in a more physiologically relevant system, we next investigated whether erythrocytes could be protected from lysis in classical and alternative pathway assays. To analyze the impact on classical pathway-mediated lysis, sheep red blood cells were sensitized with polyclonal rabbit anti-sheep Abs (CH50, **Supplementary Figure 7C**). These cells were biotinylated and treated with bsAbs (anti-FH/C4BP x anti-biotin). To analyze the impact on the alternative pathway, rabbit red blood cells (which lack endogenous FH binding) were biotinylated and treated with bsAbs (anti-FH/C4BP x anti-biotin), (AP50, **Supplementary Figure 7C**).

**Figure 4:**
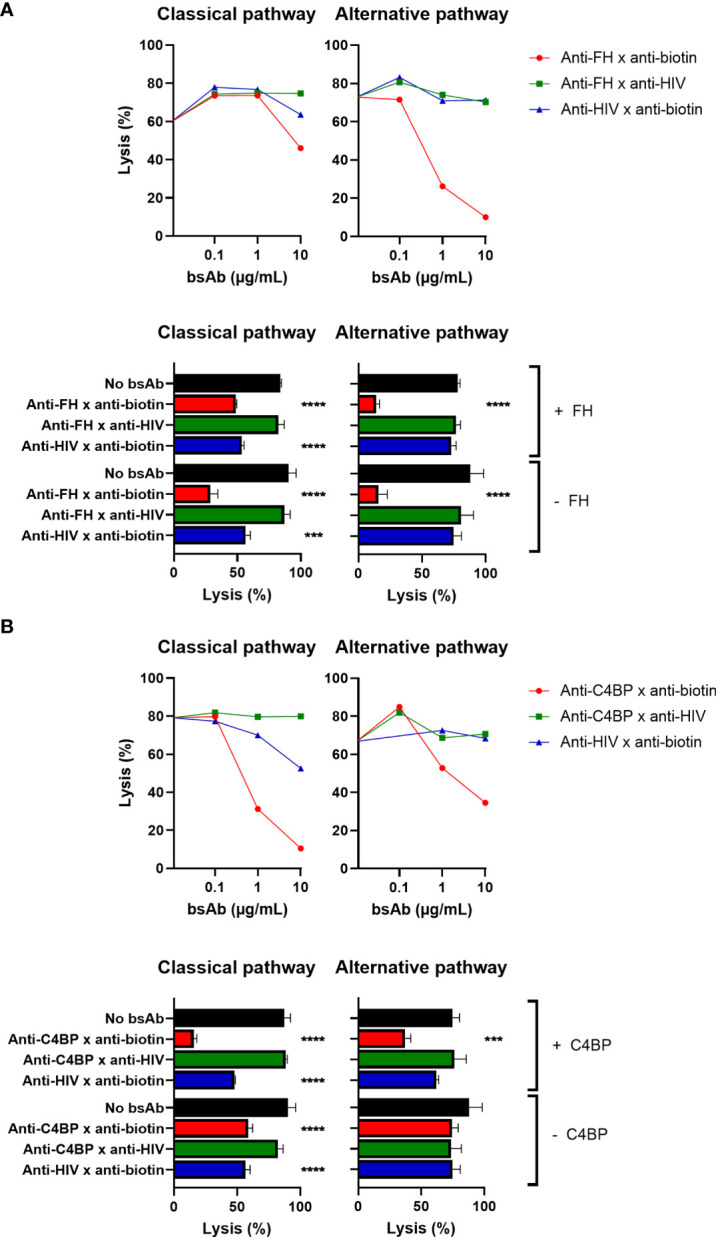
Targeted bispecific antibodies protect erythrocytes from complement-mediated lysis. The effect of the targeted bsAbs on activity of the classical pathway was analyzed in a hemolytic assay. Biotinylated antibody-sensitized sheep red blood cells were incubated with anti-FH x anti-biotin **(A)** or anti-C4BP x anti-biotin **(B)** or control bsAbs, with (+) or without (-) exogenous FH/C4BP. Subsequently, cells were incubated with 0.5% NHS and centrifuged, and supernatants were measured. The effect of the targeted bsAbs on activity of the alternative pathway was analyzed in a similar assay. Biotinylated rabbit red blood cells were incubated with anti-FH x anti-biotin **(A)** or anti-C4BP x anti-biotin **(B)** or control bsAbs, with (+) or without (-) exogenous FH/C4BP. Subsequently, cells were incubated with 5% NHS and centrifuged, and supernatants were measured. Bars indicate means and error bars indicate standard deviations of one representative experiment out of at least three experiments at a concentration of 10ug/ml. One-way ANOVA compared to no bsAb, ***P < 0.001; ****P < 0.0001.

Upon incubation with NHS, the bsAb binding both FH and biotin clearly inhibited lysis of red blood cells, whereas the control bsAbs did not (**Figure 4A**). We observed a decrease in lysis from 90% for the control to only 28% for the anti-FH bsAb. Since FH is the main fluid-phase regulator of the alternative pathway, it is not surprising that the effect on alternative pathway-mediated lysis is more pronounced than the effect on classical pathway-mediated lysis, where the observed effect is likely related to inhibition of the alternative pathway acting as the amplification loop of the classical pathway. As seen before for the plate-bound assays, adding exogenous FH to NHS was not necessary and did not improve the complement-inhibiting effect.

Similarly, the bsAb binding both C4BP and biotin (and not the control bsAbs) also clearly decreased complement-mediated lysis of red blood cells (**Figure 4B**). We observed a decrease in lysis from 87% for the control to only 16% for the anti-C4BP bsAb in the optimal conditions. Since C4BP is the main fluid-phase regulator of the classical pathway, it was to be expected that the effect on classical pathway-mediated lysis would be larger than the effect on alternative pathway-mediated lysis. For the classical pathway, adding exogenous C4BP to NHS was not necessary, but did improve the complement-inhibiting effect, as shown for the plate-bound assays but in contrast to the FH results. However, for the alternative pathway, adding exogenous C4BP to NHS was required to observe a complement-inhibiting effect (27). Also in a completely human system, using human red blood cells, human serum, and human anti-Vel IgM Abs, the bsAb binding both C4BP and biotin clearly decreased complement-mediated lysis, and adding exogenous C4BP was not necessary (**Supplementary Figure 6**).

In summary, the bsAbs were also able to protect erythrocytes from lysis in classical and alternative pathway assays, where the FH-binding bsAb was most effective in inhibiting alternative pathway-mediated lysis and the C4BP-binding bsAb was most effective in preventing classical pathway-mediated lysis.

### Targeted bispecific antibodies protect white blood cells from complement-mediated cytotoxicity

**Figure 5:**
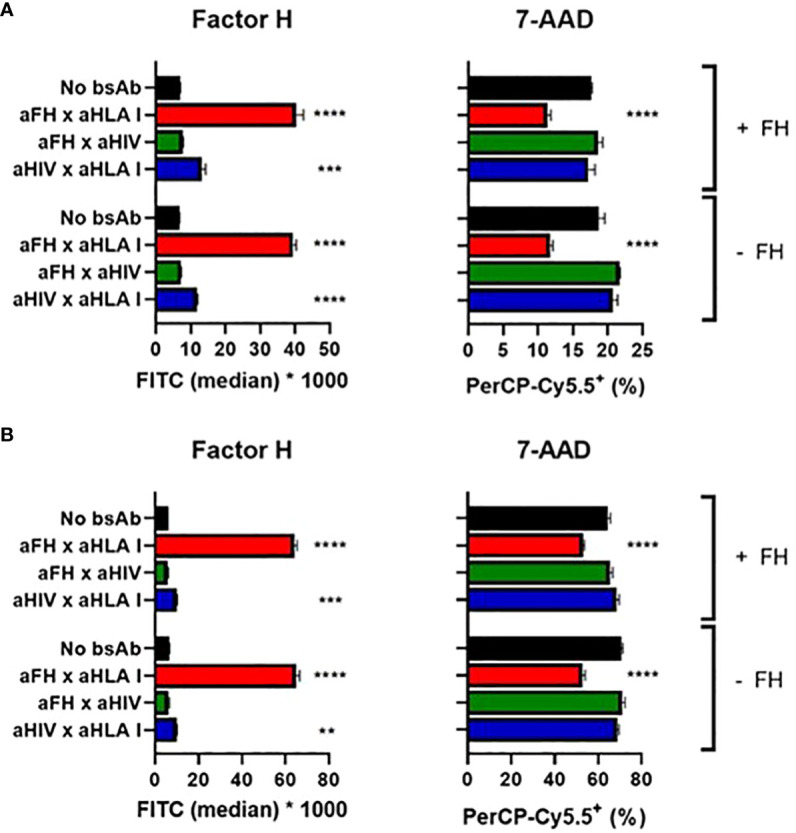
Targeted bispecific antibodies protect white blood cells from complement-mediated cytotoxicity. BsAbs were functionally tested in a complement-dependent cytotoxicity assay. **(A)** Ramos cells were incubated with anti-CD20 wild-type with or without anti-FH x anti-HLA I or control bsAbs, with (+) or without (-) exogenous FH. Cells were washed and incubated with 10% NHS. FH and cell viability (using 7-AAD) were measured. **(B)** PBMCs were incubated with anti-CD52 wild-type with or without anti-FH x anti-HLA I or control bsAbs, with (+) or without (-) exogenous FH. Cells were washed and incubated with 10% NHS. FH and cell viability (using 7-AAD) were measured. Bars indicate means and error bars indicate standard deviations of one representative experiment out of at least three experiments. One-way ANOVA compared to no bsAb, **P < 0.01; ***P < 0.001; ****P < 0.0001.

Finally, we investigated whether our bsAbs would protect human leukocytes from complement-mediated lysis. Since these cells express human membrane-bound complement inhibitors (in contrast to the previously used sheep/rabbit erythrocytes) and therefore are partly protected against complement-mediated lysis, we tested whether the protective effect of our bsAbs could still be observed for these cells. First, we used Ramos cells, a B-cell line expressing CD20, and complement-mediated lysis of these cells was triggered by incubating these cells with anti-CD20 Abs and NHS. We studied if targeted inhibition of complement activation could be achieved by using a bsAb that binds human leukocyte antigen class I (HLA I), a protein expressed on the surface of all nucleated cells. The bsAb binding both FH and HLA I was able to target FH to the cells, which resulted in protection from lysis (**Figure 5A**). As before for the plate-bound and hemolytic assays, adding exogenous FH to NHS was not necessary and did not further improve the complement-inhibiting effect.

To confirm this complement-inhibiting effect, we switched from a cell line to peripheral blood mononuclear cells (PBMCs). Since CD52 is expressed on the surface of most PBMCs, cells were sensitized with anti-CD52 Abs to activate the classical pathway of the complement system upon adding NHS. The bsAb binding both FH and HLA I was able to target FH to the cells, which resulted in protection from lysis (**Figure 5B**). Again, adding exogenous FH to NHS was not necessary and did not clearly improve the complement-inhibiting effect. In addition we verified that in the complex mixture of PBMCs the bsAb would indeed mediate a cell type specific accumulation of FH or C4BP. Indeed testing bsAb based on rituximab, so targeting B cells only we could demonstrate an accumulation of FH on B cells, but not on T cells, and similarly of C4BP on B cells and not on T cells (**Supplementary Figure 8**).

Altogether, the bsAbs locally inhibited complement activation initiated by the classical, lectin, and alternative pathway in plate-bound assays. The bsAbs also protected liposomes from complement-mediated lysis as well as erythrocytes in classical and alternative pathway assays. Finally, the bsAbs protected human leukocytes from complement-mediated lysis.

## Discussion

In this study we tested a novel concept of locally targeted complement inhibition using a format of bsAbs enforcing colocalization of human endogenous complement regulators and specific (auto)antigens. Therefore, we designed a comprehensive set of bsAbs where one arm binds to a site-specific target and the other arm binds to the endogenous complement regulators FH or C4BP, and tested whether they could provide locally targeted complement inhibition. We indeed observed that the bsAbs inhibited complement activation in plate-bound assays, but also in liposome and cellular assays, protecting liposomes, erythrocytes, as well as lymphocytes (for the anti-FH bsAb) leukocytes from complement-mediated lysis. Taken together, this demonstrates that we can achieve targeted complement inhibition using engineered bsAbs that bind local antigens and endogenous complement regulators.

We compared our bsAbs, binding both a site-specific target (e.g., DNP) and a complement regulator (FH or C4BP), with control bsAbs binding only the target or the complement regulator and with the other arm targeting an irrelevant antigen (HIV gp120). In most assays the control bsAb binding only the target also resulted in some inhibition of complement activation, which was not associated with an increase in bound FH or C4BP. This might be explained by partial shielding of complement-activating molecules in the plate-bound complement activation assays (e.g. due to steric hindrance with the control antibody), thereby reducing their capability for complement activation. The fact that this phenomenon is not observed for the alternative pathway indeed agrees with this notion, since in the alternative pathway spontaneous C3 cleavage occurs, and activation is not dependent on an initiating molecule that can be shielded. Importantly, the inhibition of complement activation was always stronger with the bsAb binding both the target and the complement regulator.

The few complement-inhibitory drugs that have made their way into the clinic, and were approved for the treatment of several complement-mediated diseases, inhibit complement systemically, which is associated with important downsides including the increased risk for infections and need for high dosing. Eculizumab (anti-C5 antibody) is an example of an FDA- and EMA-approved complement-inhibitory drug to treat patients suffering from paroxysmal nocturnal hemoglobinuria, atypical hemolytic uremic syndrome, generalized myasthenia gravis, and neuromyelitis optica spectrum disorder (28). Since life-threatening and fatal meningococcal infections have occurred in patients treated with eculizumab, patients are immunized with meningococcal vaccines prior to administering eculizumab, and patients are monitored closely for early signs of meningococcal infections. Therefore, eculizumab is not broadly applicable and only used in patients with rare diseases, making this drug one of the most expensive immunotherapeutics.

Locally targeted complement inhibition conceivably would not suffer from these limitations (increased risk for infections, need for high dosing, and extreme costs) and would therefore be highly desirable. Hence, in this study we aimed to achieve local inhibition of the complement system by using bsAbs. Whether this approach indeed does not increase the risk for infections should be confirmed *in vivo*. However, our observation that the concentrations of endogenous FH and C4BP in 1% serum are sufficient to mediate local inhibition suggests that our bsAbs would not deplete the regulators from the circulation when applying bsAbs *in vivo*. If necessary, patients could be treated with a bsAb and an exogenous regulator. Since the bsAbs bind to local antigens and therefore result in targeted inhibition, lower dosing and thereby lower costs are expected to be additional advantages. Importantly, in locations with high serum concentration there can conceivably be more complement activation than at locations with lower complement levels, but importantly in our approach the levels of the endogenous complement inhibitors parallel the level of complement, providing adequate inhibition.

Targeted complement activation using bispecific molecules has been studied extensively in the battle against cancer and infections. For example, Pedersen and colleagues generated bispecific nanobodies binding properdin and the validated cancer antigen EGFR, and showed that these bispecific molecules were able to activate complement on EGFR-expressing cancer cells (29). In addition, immunoconjugates binding a positive regulator of the alternative pathway (FHR4) and the HER2 cancer antigen have been shown to induce complement-dependent cytotoxicity to HER2-expressing tumor cells more efficiently than anti-HER2 Abs (30). Cruz and colleagues used bsAbs binding the complement-initiating protein C1q and cell surface targets expressed by bacteria or tumor cells and showed efficient killing of these cells (31). Also smaller bi-specific antibodies, called (BiCEs) that target both C1q and EGFR have been successfully applied (32). Furthermore, bsAbs binding tumor antigens and membrane-bound complement inhibitors (e.g., Crry or CD55) have been shown to induce killing of tumor cells by blocking these inhibitors specifically on tumor cells (33, 34). These studies show that targeted complement activation in the context of cancer and infections is feasible.

Regarding targeted complement inhibition, Alawieh and colleagues generated a fusion protein targeting a post-stroke neoepitope and Crry, an ortholog of the human complement inhibitor CR1, and showed inhibition of neuroinflammation after stroke in mice (35). Similarly, Werneburg and colleagues also used Crry, but fused it to a domain of CR2, which binds activated C3, in the context of multiple sclerosis (36). However, local injections in the eye were given to achieve site-specific complement inhibition. Crry is a murine protein, so the abovementioned fusion proteins cannot be used in humans. However, a similar fusion protein has been designed containing domains of CR2 and the human protein CR1 (TT32 (37)), targeting this complement inhibitor to sites where complement activation has occurred. The same group also generated a CR2-FH fusion protein (TT30 (38)). Whereas these approaches can achieve complement inhibition at sites where complement activation has occurred, for example in arthritis, this can also happen on C3b-coated bacteria, increasing infectious risk. With our study, we present an approach to achieve site-specific targeted human complement inhibition using bispecific molecules without the need of local administration.

Although not addressed directly in this study, additional advantages of using endogenous complement inhibitors include the non-immunogenic nature as well as the fact that endogenous inhibitors, such as FH and C4BP, can perform their inhibitory function multiple times, both regarding cofactor activity towards FI as well as the decay-accelerating activity, since the affinities for C3b and C4b are relatively low (14). This is in sharp contrast to immunogenic constructs that artificially combine domains from different molecules, or the complement-depleting nature of the currently used anti-complement biologicals.

The approach presented in this study is very broadly applicable. The current work is primarily focused on model antigens, like DNP, Biotin and HLA to target the bispecific antibodies to a particular surface. The data show that such targeting indeed provides sufficient endogenous inhibitors to affect local complement inhibition. We choose three different model antigens to showcase that this approach is broadly applicable and not limited to a single antigen. The observation that the targeted inhibition approach using bsAb targeting three unrelated model-antigens works gives confidence that it will also work on additional tissue-specific antigens. Our results show that the C4BP-targeting bsAbs do not inhibit activation of the alternative pathway, in line with the fact that the alternative pathway does not involve C4b. Therefore, depending on whether the disease is more classical or lectin pathway-mediated or alternative pathway-mediated, either C4BP or FH could be targeted, respectively. The other arm can be designed to bind specific (auto)antigens, for example collagen in arthritis (2), targeting the regulator to the joints, locally inhibiting complement activation. In nephritis, the glomerular basement membrane could be targeted (3), bringing the regulator to the kidneys, and in myasthenia gravis, the acetylcholine receptor can be targeted (4), causing the regulator to locate to the neuromuscular junction. A final example is transplant rejection, in which a transplanted organ that is not fully HLA-matched elicits an immune response (5). In this case, the transplanted organ could be treated with the bsAb binding to FH or C4BP and HLA, shielding the antigenic epitopes, but also targeting a complement regulator to the transplanted organ, protecting the organ from complement-mediated attack.

Altogether, we show in this study that we can achieve targeted complement inhibition using engineered bsAbs that bind local antigens and endogenous complement regulators, which is promising for many clinical conditions such as autoimmune diseases and transplant rejection. Future *in vivo* studies are necessary to investigate the reduced risk for infections and the effectiveness of our bsAbs in animal models of autoimmune diseases and transplant rejection.

## Data availability statement

The original contributions presented in the study are included in the article/**Supplementary Material**. Further inquiries can be directed to the corresponding author.

## Ethics statement

Ethical approval was not required for the studies involving humans because Complement active human serum was obtained through the Leiden University Medical Center Voluntary Donor Service (LuVDS) under protocol number L21.036. The studies were conducted in accordance with the local legislation and institutional requirements. Written informed consent to participate in this study was not required from the participants or the participants’ legal guardians/next of kin in accordance with the national legislation and the institutional requirements. Ethical approval was not required for the studies on animals in accordance with the local legislation and institutional requirements because only commercially available established cell lines were used.

## Author contributions

HW: Data curation, Methodology, Validation, Investigation, Visualization, Software, Writing – review & editing. FB: Writing – original draft, Writing – review & editing. DD: Writing – review & editing. LA: Writing – review & editing. NB: Writing – review & editing. JP: Writing – review & editing. RZ: Writing – review & editing. CG: Writing – review & editing. KG: Writing – review & editing. TD: Writing – review & editing. GV: Writing – review & editing. AB: Writing – review & editing. ED: Writing – review & editing. PP: Writing – review & editing. TS: Writing – review & editing. LT: Writing – original draft, Writing – review & editing.
