# Supplementary material for: Targeted complement inhibition using bispecific antibodies that bind local antigens and endogenous complement regulators
Source: Front Immunol. 2024 May 16;15:1288597. doi: 10.3389/fimmu.2024.1288597 (PMC11137741; doi:10.3389/fimmu.2024.1288597)
Supplement: Supplementary file 1 [file DataSheet_1.docx]

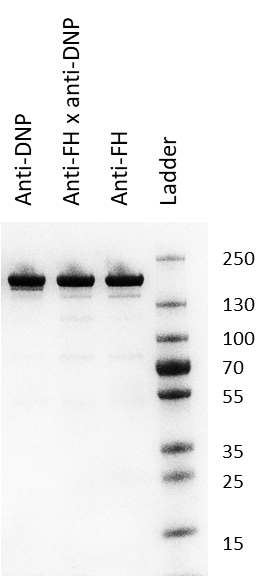


**Supplementary Figure 1.** Purity of parental and bispecific antibodies. Gel electrophoresis of 5 µg parental (anti-DNP or anti-FH) or anti-FH x anti-DNP bsAb was done using precast 4-15% Tris-Glycine gels and visualization with Coomassie Blue (InstantBlue). Ladder is in kDa.


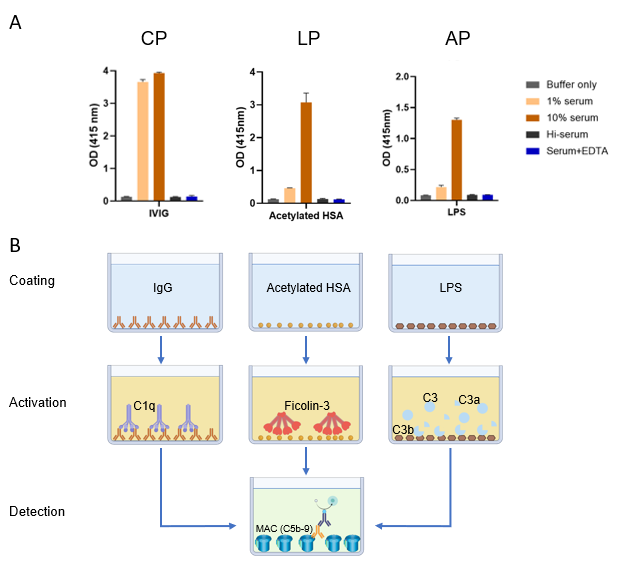


**Supplementary Figure 2.** Complement activation of classical pathway (CP), lectin pathway(LP) and alternative pathway(AP). IgG, acetylated HAS and LPS was coated the plate, to activate different pathways specifically. For the AP, serum was diluted in RPMI+MgEGTA, the other two were in RPMI. C5b-9 was used to assess the activation(A). The scheme of the ELISA experiment was shown as B.


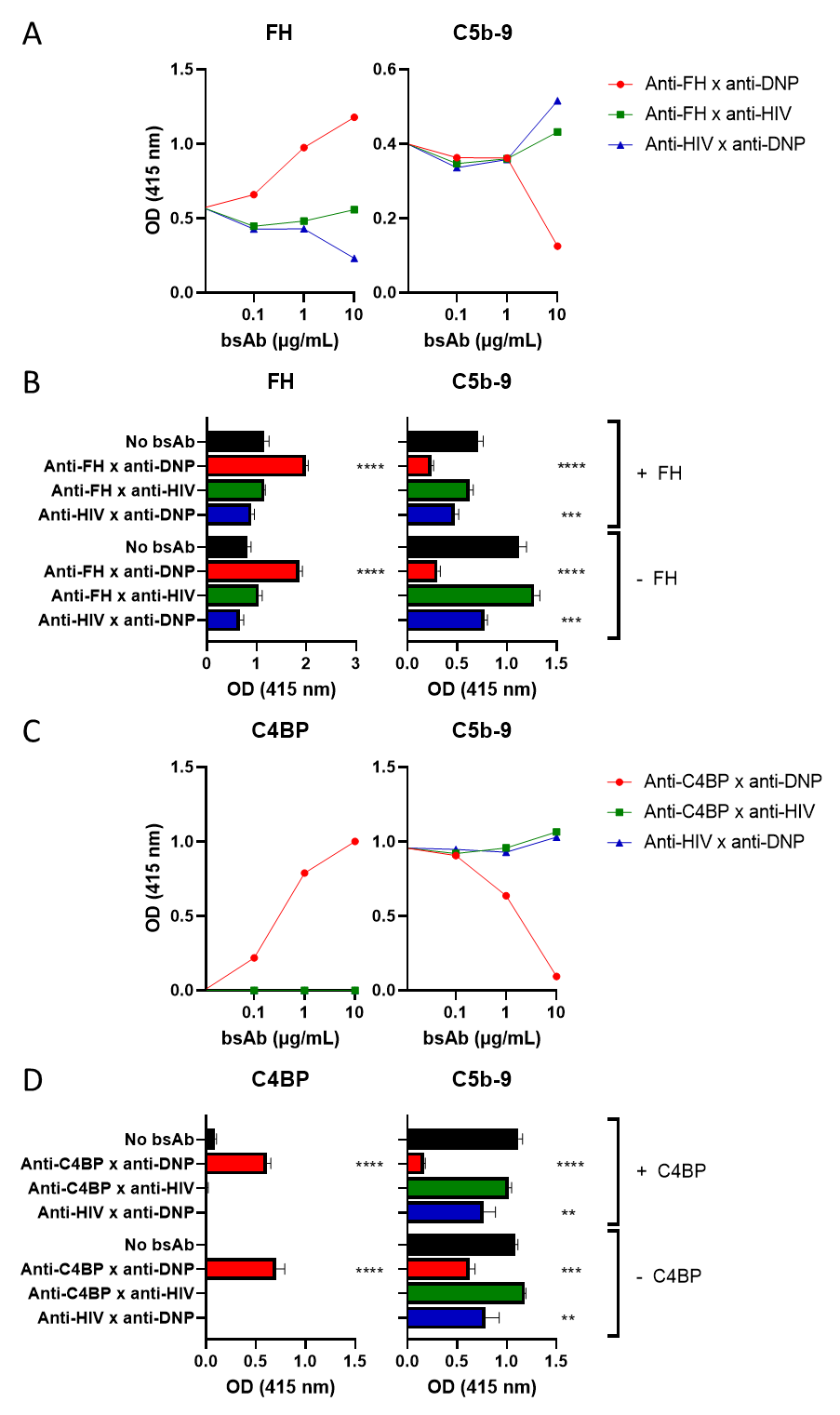


**Supplementary Figure 3.** Targeted bispecific antibodies that bind endogenous complement inhibitors decrease IgM-initiated complement activation. BsAbs were functionally tested in plate-bound complement activation assays. Plates coated with BSA-DNP and IgM were incubated with a titration of anti-FH x anti-DNP **(A)**, 5 µg/mL anti-FH x anti-DNP **(B)**, a titration of anti-C4BP x anti-DNP **(C)**, or 5 µg/mL anti-C4BP x anti-DNP **(D)** or control bsAbs, with NHS with (+) or without (-) exogenous FH **(B)** or C4BP **(D)**. FH **(A,B)**, C4BP **(C,D)**, and C5b-9 **(A-D)** were detected. Bars indicate means and error bars indicate standard deviations of one representative experiment out of at least three experiments. One-way ANOVA compared to no bsAb, ** P < 0.01; *** P < 0.001; **** P < 0.0001.


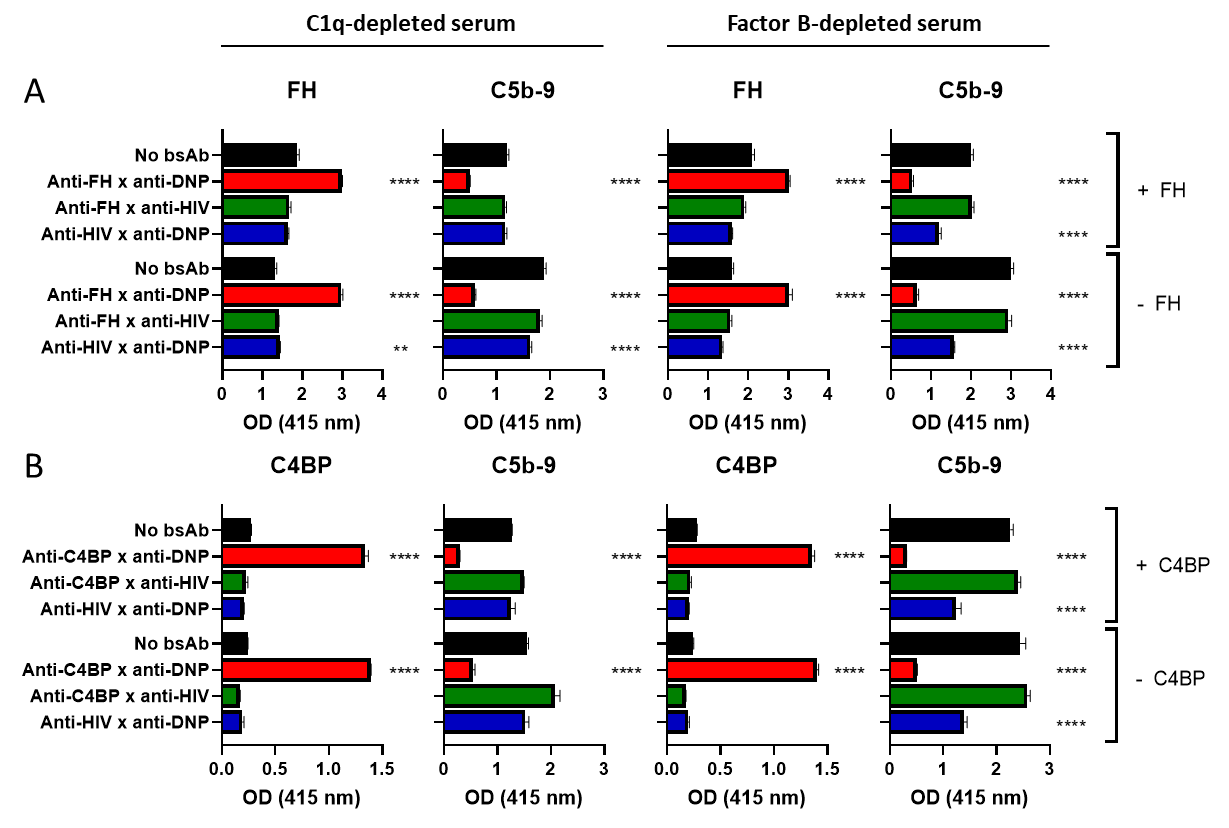


**Supplementary Figure 4.** Targeted bispecific antibodies that bind endogenous complement inhibitors decrease lectin pathway-initiated complement activation. BsAbs were functionally tested in plate-bound complement activation assays. Plates coated with BSA-DNP and acetylated HSA (lectin pathway) were incubated with 5 µg/mL anti-FH x anti-DNP **(A)** or anti-C4BP x anti-DNP **(B)** or control bsAbs, with C1q-depleted serum (left) or factor B-depleted serum (right) with (+) or without (-) exogenous FH **(A)** or C4BP **(B)**. FH **(A)**, C4BP **(B)**, and C5b-9 **(A,B)** were detected. Bars indicate means and error bars indicate standard deviations of one representative experiment out of at least three experiments. One-way ANOVA compared to no bsAb, ** P < 0.01; **** P < 0.0001.


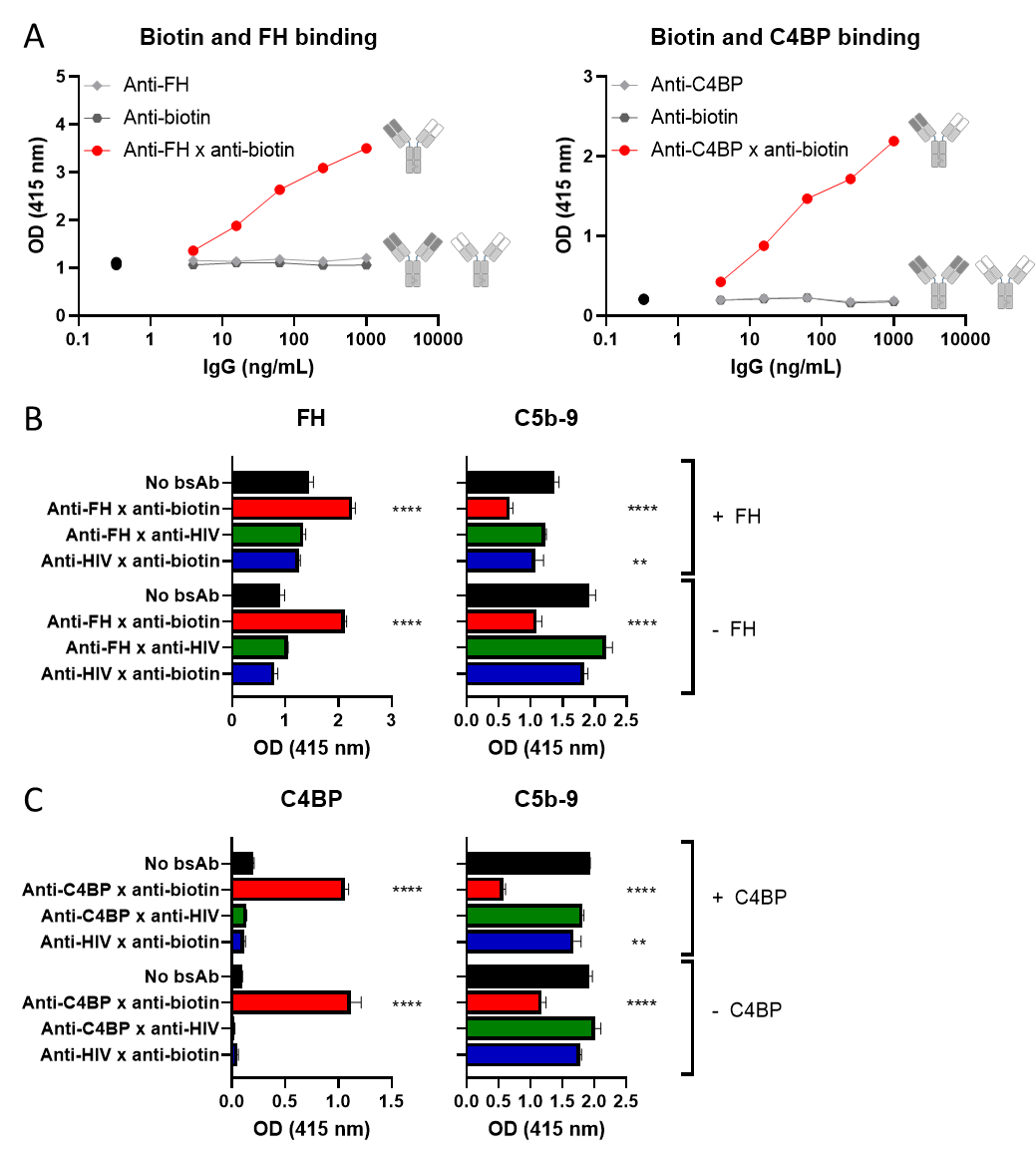


**Supplementary Figure 5.** Biotin-targeting bispecific antibodies that bind endogenous complement inhibitors decrease complement activation. **(A)** BsAbs were tested for simultaneous binding to both antigens in a bispecificity ELISA. Plates were coated with biotinylated BSA-DNP and incubated without Abs (black circle) or with anti-FH (left, light gray diamonds), anti-C4BP (right, light gray diamonds), anti-biotin (dark gray hexagons) parental Abs, or bsAbs (red circles). FH or C4BP was added and these inhibitors were detected. **(B)** BsAbs were functionally tested in plate-bound complement activation assays. Plates coated with biotinylated BSA-DNP and IgG (classical pathway) were incubated with 5 µg/mL anti-FH x anti-biotin **(B)** or anti-C4BP x anti-biotin **(C)** or control bsAbs, with NHS with (+) or without (-) exogenous FH **(B)** or C4BP **(C)**. FH **(B)**, C4BP **(C)**, and C5b-9 **(B,C)** were detected. Bars indicate means and error bars indicate standard deviations of one representative experiment out of at least three experiments. One-way ANOVA compared to no bsAb, ** P < 0.01; **** P < 0.0001.

**Supplementary Figure 6.** Targeted bispecific antibodies protect human erythrocytes from complement-mediated lysis. The effect of the targeted bsAbs on activity of the classical pathway was analyzed as in Figure 4 but in a completely human system using bromelin-treated biotinylated human red blood cells, 5% serum, and anti-Vel IgM. Bars indicate means and error bars indicate standard deviations of one representative experiment out of at least three experiments. One-way ANOVA compared to no bsAb, ** P < 0.01.


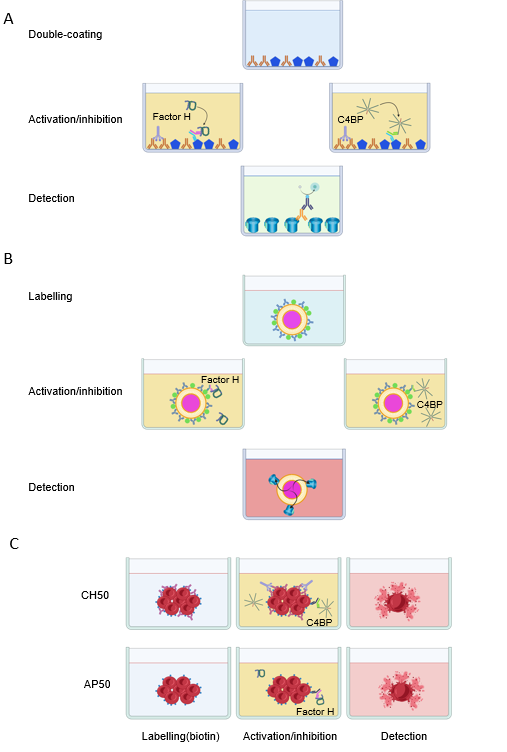


**Supplementary Figure 7.** The schematic overview of the experiments used in this paper. Double-coating ELISA to test the complement inhibition function of bsAbs (A). Liposome lysis experiment to test the function of bsAbs (B). Erythrocytes lysis experiment to test the function of bsAbs (C).


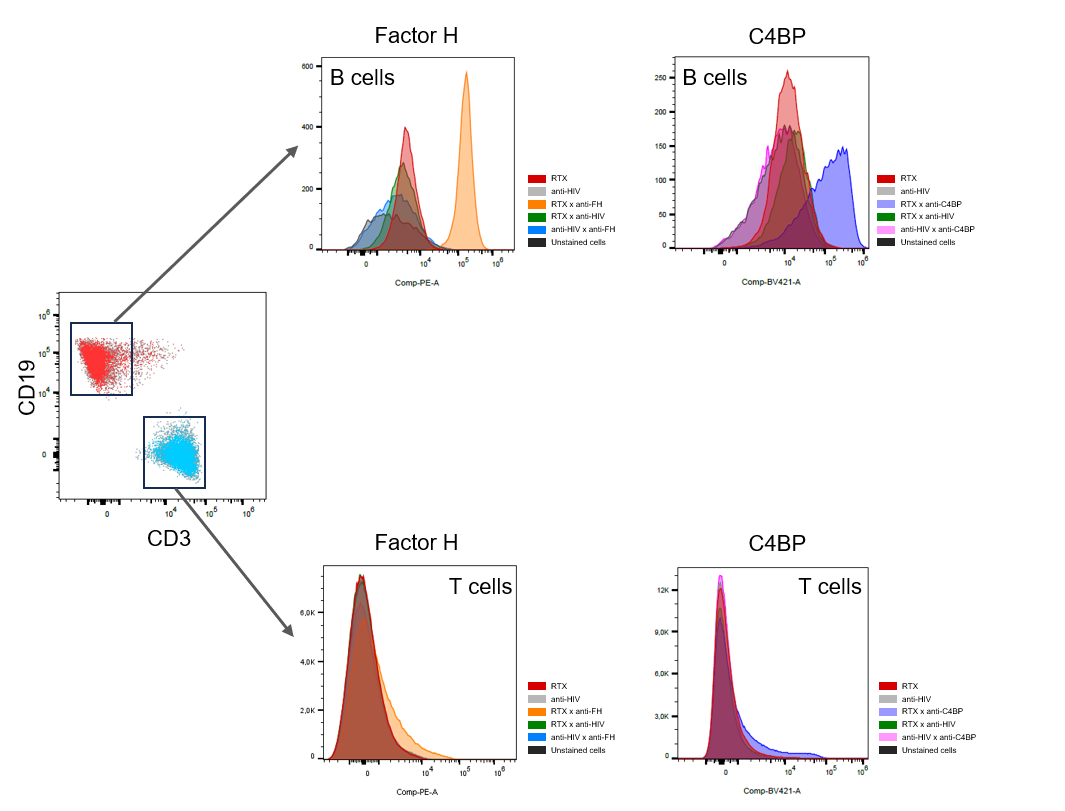


**Supplementary Figure 8**. Flow cytometry experiment to verify presence of FH and C4BP on specific cell populations in PBMCs.

Isolated PBMCs were incubated with bsAbs targeting CD20 (Rituximab, RTX) and FH or C4BP or relevant controls. Next PBMCs were incubated 20% normal human serum following washing the presence of FH or C4BP, was established by staining cells for the presence of FH or C4BP. And staining for cell specific markers CD3 and CD19, to identify T cells and B cells respectively. Data show a specific accumulation of FH or C4BP only on the cells targeted (B cells) and not on the control cells (T cells).
